# Pharmaceutical quality of seven generic Levodopa/Benserazide products compared with original Madopar® / Prolopa®

**DOI:** 10.1186/2050-6511-14-24

**Published:** 2013-04-23

**Authors:** Urs E Gasser, Anton Fischer, Jan P Timmermans, Isabelle Arnet

**Affiliations:** 1 ClinResearch Ltd, Aesch, Switzerland; 2 F. Hoffmann-La Roche Ltd, Basel, Switzerland; 3 Pharmaceutical Care Research Group, Department of Pharmaceutical Sciences, University of Basel, Klingelbergstr. 50, Basel, 4056, Switzerland

**Keywords:** Parkinson’s Disease, Levodopa, Benserazide hydrochloride, Pharmaceutical quality, Generics

## Abstract

**Background:**

By definition, a generic product is considered interchangeable with the innovator brand product. Controversy exists about interchangeability, and attention is predominantly directed to contaminants. In particular for chronic, degenerative conditions such as in Parkinson’s disease (PD) generic substitution remains debated among physicians, patients and pharmacists. The objective of this study was to compare the pharmaceutical quality of seven generic levodopa/benserazide hydrochloride combination products marketed in Germany with the original product (Madopar® / Prolopa® 125, Roche, Switzerland) in order to evaluate the potential impact of Madopar® generics versus branded products for PD patients and clinicians.

**Methods:**

Madopar® / Prolopa® 125 tablets and capsules were used as reference material. The generic products tested (all 100 mg/25 mg formulations) included four tablet and three capsule formulations. Colour, appearance of powder (capsules), disintegration and dissolution, mass of tablets and fill mass of capsules, content, identity and amounts of impurities were assessed along with standard physical and chemical laboratory tests developed and routinely practiced at Roche facilities. Results were compared to the original “shelf-life” specifications in use by Roche.

**Results:**

Each of the seven generic products had one or two parameters outside the specifications. Deviations for the active ingredients ranged from +8.4% (benserazide) to −7.6% (levodopa) in two tablet formulations. Degradation products were measured in marked excess (+26.5%) in one capsule formulation. Disintegration time and dissolution for levodopa and benserazide hydrochloride at 30 min were within specifications for all seven generic samples analysed, however with some outliers.

**Conclusions:**

Deviations for the active ingredients may go unnoticed by a new user of the generic product, but may entail clinical consequences when switching from original to generic during a long-term therapy. Degradation products may pose a safety concern. Our results should prompt caution when prescribing a generic of Madopar®/Prolopa®, and also invite to further investigations in view of a more comprehensive approach, both pharmaceutical and clinical.

## Background

Parkinson’s disease (PD) is a progressive neurodegenerative disorder affecting primarily dopaminergic neuronal systems. Typical manifestations include motor symptoms (bradykinesia, rigidity, tremor, gait and postural instability) and non-motor symptoms including cognitive and emotional dysfunction [1-3]. Levodopa or L-dopa is a naturally occurring amino acid (L-3,4-dihydroxyphenylalanine). It is a prodrug and the precursor of dopamine (DA), a neurotransmitter that is severely reduced in PD due to degeneration of neuronal cells [2,3]. Levodopa has been the standard medical therapy for PD since its discovery approximately 40 years ago [4] and is recognized as a classic example of a brain neurotransmitter substitution therapy. When given systemically, levodopa crosses the blood–brain barrier and is converted to DA by L-dopa decarboxylase (DDC) [5]. When administered orally, a high pre-systemic conversion to DA occurs in the gut by the enzyme L-amino acid decarboxylase (AADC), reducing the systemic available dose of levodopa to 30% [6,7]. Levodopa is thus coadministered in a 4/1 ratio with an AADC inhibitor such as benserazide hydrochloride. This combination can triple the oral bioavailability of levodopa, and markedly reduces both the required levodopa therapeutic dose and the severity of dopamine-mediated gastrointestinal and cardiovascular side-effects [8]. Levodopa has a short half-life of 1.5 h, even when coadministered with an AADC inhibitor [9]. When administered with benserazide hydrochloride, the initial dose is 100 mg to 200 mg levodopa daily given as levodopa 50 mg/benserazide 25 mg up to 2 to 4 times a day. Treatment may start at levodopa 300 mg daily in advanced disease. The recommended maximum maintenance dose is 800 mg levodopa daily in multiple divided doses [9,10].

Levodopa is a white or almost white crystalline powder which darkens on exposure to air and light [10]. It is odourless, almost tasteless, slightly soluble in water and soluble in aqueous solutions of mineral acids and alkali carbonates [10]. Levodopa is purchased on the pharmaceutical market. Benserazide hydrochloride is a white or almost white crystalline powder, slightly soluble in ethanol, and soluble in water. Benserazide hydrochloride decomposes slowly in aqueous solution. It is synthetically produced by Roche.

Madopar®/Prolopa® 125 mg (levodopa 100 mg + benserazide hydrochloride 25 mg) exists as scored tablets and capsules for Parkinson’s disease [9].

By definition, a generic product is considered interchangeable with the innovator brand product and needs to demonstrate the same qualitative and quantitative composition in active substances, the same pharmaceutical form and bioequivalence with the reference product after a single dose [11]. The different salts, esters, complexes or derivatives of an active substance are considered to be the same active substance, and thus different excipients, colour agents, flavours and preservatives are allowed. Generics may also differ in characteristics such as shape, size, colour, scoring configuration, and release mechanisms [12-16]. Controversy exists about interchangeability, mainly because of the questionable validity of the current criteria the evaluation is performed in a small, young and healthy population; no clinical efficacy data are required; short term study [17]. Thus, therapeutic equivalence is not necessarily guaranteed. However, attention is predominantly directed to contaminants that could cause adverse clinical events and ultimately fatal issues [5-7]. In clinical settings, health professionals are aware that patient response and susceptibility to levodopa vary widely, especially in advanced PD, and that levodopa blood levels correlate with the emergence of many symptoms, including motor manifestations like dyskinesia and “off” periods. Thus, even small variations in levodopa availability and consequently subtle fluctuations in levodopa blood levels can trigger motor complications. Since generic formulations differ from the branded product mainly in their excipients, which may affect absorption and bioavailability [15], simple bioequivalence cannot suffice to ensure comparable clinical efficacy and safety, especially in PD.

Roche’s standard operating procedures (SOPs) describe the qualitative and quantitative pharmaceutical tests for physical and chemical purity of the substance. They were accepted by regulatory authorities as part of the Madopar® registration documentation and form the basis of Mapopar®/Prolopa® specifications in the United States, European and British Pharmacopoeias. The current investigation compares the pharmaceutical quality of seven generic products with the specifications of the original Madopar® and raises questions about safety and interchangeability.

## Methods

Samples of generic levodopa/benserazide hydrochloride products, all 100 mg/25 mg formulations, were purchased in 2011 as commercial goods in a community pharmacy in Germany and tested within their expiry dates.

### Pharmaceutical quality tests

The tests were colour (tablets and capsules), colour and appearance of powder (capsules), mass of tablets and fill mass of capsules, disintegration and dissolution, content of active pharmaceutical ingredient (API), and the identity and amounts of impurities.

### Colour

Tablet colour was assessed visually according to the standards described in the “Munsell Book of Color” (1996). Capsule colour was assessed by comparison to reference capsules. Madopar® specifications for colour are pale red for tablets and flesh-coloured opaque for the capsule body and powder-blue opaque for the capsule cap.

### Mass of tablets

Twenty tablets were weighed and average mass was calculated. Madopar® specifications for average mass of tablets are 267–283 mg (275 mg ± 3%).

### Fill mass of capsules

Twenty capsules were weighed, emptied, and the empty capsules weighed; the average weight of the capsule fill mass was derived from the difference. Madopar® specifications for capsule fill mass are 142.5-157.5 mg (150 mg ± 5%).

### Disintegration time

Tablets and capsules were tested according to Ph. Eur. / USP (tablets: apparatus A without discs using water at 37°C; capsules: apparatus A using discs and 0.1 M HCl at 37°C). Madopar® specifications for disintegration time are not more than 15 min for tablets and not more than 30 min for capsules.

### Dissolution

Dissolution was tested using the Ph. Eur. rotating basket apparatus and 0.1 N HCl at 37°C as the dissolution medium. Samples were measured by UV-HPLC at 220 nm. Madopar® shelf-life specifications for dissolution after 30 min are at least 75% for tablets and at least 80% for capsules.

### Content of levodopa, benserazide hydrochloride, related substances and impurities

Madopar® specifications for content of active substances are levodopa 95.0-105.0 mg (100 mg ± 5%) and benserazide hydrochloride 27.1-29.9 mg (28.5 mg ± 5%) per unit. The content of active substances and degradation products was determined using HPLC. Benserazide is degradated by hydrolysis to Ro 04–1419, which can bind one molecule of benserazide to the dimer Ro 08–1580. The specifications for content of specific degradation products of benserazide hydrochloride are limited to 3.49% (tablets) and 0.49% (capsules). The maximum permissible amount of other impurities is set at 1.04% (tablets) and 0.54% (capsules). The specifications for other impurities are no more than 0.54% (tablets) and 0.24% (capsules) for any individual impurity.

## Results

Analysis was performed according to the standard physical and chemical laboratory tests developed and routinely practiced at Roche facilities.

### Physical characteristics

The physical characteristics of the tested products are shown in Table 1. None of the generic tablets was cross-scored on both sides like the original product, but all had either a single break bar on both sides (Betapharm) or on one side only. The colours of all generic products were similar to Madopar®/Prolopa® except for one capsule formulation (Teva).

**Table 1:** Physical characteristics of Madopar®/Prolopa® and seven generic products

**Brand name**	**Manufacturer**	**Colour**	**Appearance**	
**Tablets**				
Madopar	Roche Pharmaceuticals	Pale red	Above: cross-scored	
			Below: cross-scored	
Levodopa/ Benserazid beta	Betapharm Arzneimittel GmbH	Pale red, speckled	Above: score line	
			Below: score line	
Levodopa/ Benserazid-CT	CT Arzneimittel GmbH	Pale red, speckled	Above: score line	
			Below: none	
dopadura B	MYLAN dura	Pale red, speckled	Above: score line	
			Below: none	
Levodopa/ Benserazid ratiopharm	ratiopharm GmbH	Pale red, strongly speckled	Above: score line	
			Below: none	
**Capsules**			**Appearance of the content**	**Colour of the content**
Madopar	Roche Pharmaceuticals	Body: flesh-coloured, opaque	Fine granular powder	Light beige
		Cap: powder-blue, opaque		
Levopar	HEXAL AG	Body: flesh-coloured, opaque	Fine granular powder	Brown
		Cap: powder-blue, opaque		
Levodopa comp. B STADA	STADApharm GmbH	Body: flesh-coloured, opaque	Fine granular powder	Almost white
		Cap: powder-blue, opaque		
Levobens-Teva	TEVA GmbH	Body: midnight blue, opaque	Fine granular powder	Almost white
		Cap: fluorescent-pink, opaque		

### Mass, fill mass, disintegration time and dissolution rate

Mass exceeded the upper limits of the specifications in five of the seven generic products (Table 2), and by almost 200% for all three capsule formulations. Disintegration times (min - max: tablets 4.30 - 6.30 min; capsules 5.40 -16.10 min) were within specification. Dissolution values after 30 min complied with the specifications, however, some single values were below specification (Levobens-Teva 16%, 25%; Levopar 68%, 49%, 72% 78%), indicating large variations in dissolution properties for single capsules.

**Table 2:** Mass, content of active ingredients, and dissolution time of seven generic products compared to Madopar®/Prolopa® specifications

**Brand name**	**Mass (mg)**	**Levodopa (mg)**	**Benserazide hydrochloride (mg)**	**Dissolution after 30 min**
**Tablet specifications**	**267.0-283.0**	**95.0-105.0**	**27.1-29.9**	**>75%/ >75%**
Levodopa/ Benserazid beta	265	92.4*	30.9*	99% / 99%
Levodopa/ Benserazid-CT	284*	98.2	27.8	101% / 97%
dopadura B	283	94.4*	26.9*	100% / 97%
Levodopa/ Benserazide ratiopharm	284*	95.5	28.8	101% / 98%
**Capsule specifications**	**Fill mass (mg) 142.5-157.5**	**95.0-105.0**	**27.1-29.9**	**>80%/ >80%**
Levopar	298.6*	98.3	27.5	80% / 84%
Levodopa comp. B STADA	299.0*	98.7	27.6	100% / 96%
Levobens-Teva	222.3*	99.5	28.3	89% / 86%

* Values marked with an asterisk indicate deviations from Roche specifications.

### Content of active substances, degradation products, and impurities

Content requirements for the active substances were unmet in two of the four tablet formulations, with content of levodopa below the limits, and content of benserazide hydrochloride below and above the limits (Table 2). All products contained impurities, exceeding the limits by 79% in one generic product (Table 3).

**Table 3:** Identity and amounts of related substances and impurities in seven generic products compared to Madopar®/Prolopa® specifications

**Degradation product**	**Ro 04-1419**	**Ro 08-1580**	**Ro 04-1419 + Ro 08-1580**	**Others each**	**Others total**
**Tablets Upper limits (%)**	**1.54**	**2.49**	**3.49**	**0.54**	**1.04**
Levodopa/ Benserazid beta	0.35	0.47	0.83	0.19, 0.09	0.28
Levodopa/ Benserazid-CT	0.28	0.48	0.75	0.09, 0.08	0.17
Levodopa/ Benserazide ratiopharm	0.25	0.37	0.62	0.16, 0.05	0.21
dopadura B	0.26	0.34	0.60	0.12	0.12
**Capsules Upper limits (%)**	**0.54**	**0.54**	**0.49**	**0.24**	**0.54**
Levopar	0.12	0.34	0.46	0.10	0.10
Levodopa comp. B STADA	0.16	0.32	0.49	0.11, 0.11	0.11
Levobens-Teva	0.36	0.25	0.62*	0.10, 0.12, 0.10	0.33

*Values marked with an asterisk indicate deviations from Roche specifications.

## Discussion

Comparison of the pharmaceutical quality of Madopar®/Prolopa® with seven generic products showed that at least one of the tested parameters fell outside Roche shelf-life specifications in all products tested and identified two areas of concern: Content of active substances and impurities. Since even small variations of levodopa availability and consequently subtle fluctuations in levodopa blood levels can trigger motor complications, the observed out-of-specification values could suffice to unbalance symptom management, especially in stabilized patients. In switching to a new formulation, patients may face weeks of complex retitration and further visits to their neurologists. In 2010–11 the worldwide shortage of branded Sinemet (carbidopa-levodopa), followed by shortage of its generic formulation (all formulations and all dosages), revealed the distress of the Parkinson community through the testimony of thousands of patients forced to switch to generics [18]. Patients uniformly reported a negative experience and several clinical issues, e.g. slower onset of effect, faster waning of effect, dose adjustment to compensate for the decreased medication effect, symptom exacerbation (e.g. dyskinesia), and side effects such as poorer sleep quality and increased impulsivity. No patient reported a preference for the generic version. Replacement medication was perceived as less effective, probably due to patients receiving differing generics at each renewal, thus getting fluctuations in blood levels with every new product.

The development of a branded formulation requires the assessment of pharmacokinetic parameters in healthy subjects, and a clinical study program to proof efficacy, safety and tolerability in the target patient population. Market authorisation for generic equivalents, however, requires only the documentation of bioequivalence with branded counterparts in healthy subjects, using one lot of branded product, and without accounting for country to country differences [19,20]. Some small studies [21,22] suggested that the generic formulation of carbidopa-levodopa given in a single dose to PD patients was bioequivalent to brand Sinemet. However, the same authors report clinical worsening with marked motor fluctuations in a long-term open-label study following conversion to generic carbidopa-levodopa [21]. Approval by the authorities is based on the assumption that demonstrating bioequivalence in pharmacokinetic studies in healthy volunteers suffices to demonstrate similar tolerability and efficacy in patients. Differences in the excipients used for generic formulations and the presence of impurities which may affect both the absorption and bioavailability of active ingredients in patients create a potential risk of “relative therapeutic in-equivalence” [15]. Furthermore, the intra-individual peculiarity of PD patients, such as slow absorption of the first orally administered dose of medication in the morning due to low gastric motility [23] makes the variability of blood concentrations from generic drugs unpredictable. Furthermore, PD patients often use multiple drugs e.g., dopamine agonists, monoamine oxydase inhibitors, anticholinergics, and psychotropics. Since issues regarding drug-drug interactions are not addressed by bioequivalence studies, potential risks cannot be excluded [1,23]. Unpredictable blood concentrations also expose patients to a higher risk of concentration-dependent drug-drug interactions. In addition, unknown excipients and impurities may trigger allergic reactions or even intolerance [24,25]. Score lines are a further area of concern. Madopar®/Prolopa® is cross-scored for easy splitting, since many patients commonly split their tablets to adjust their doses. Easy splitting of the medication for optimal individual dosing is a critical patient-care requirement. Without score lines, or with only one score line on one side, as in three of the test generic tablets, tablet splitting usually requires the use of a sharp blade, which is a clear safety issue for a person with PD.

## Conclusions

In conclusion, these results demonstrate areas of concern in the pharmaceutical quality of generic products, such as the content of active substances, and the composition and amount of impurities. The potential risks of “relative therapeutic in-equivalence,” drug-drug interactions, and allergic reactions or intolerance should prompt caution when prescribing a generic product of Madopar®/Prolopa®, in particular in highly susceptible PD patients with co-morbidities requiring comedication. We recommend considering the substitution of Parkinson’s medication as a change in medication, requiring guidance and supervision by the patient’s physician. Any generic can be used to initiate first treatment. It will be effective and less expensive than the branded product, but will need a similar process of titration until symptom control is achieved. The challenge for patient, prescribing physician and pharmacist will be to ensure that the same generic formulation is dispensed at each refill. Switching back and forth between brand and generic, or even between generics, is a recipe for problems that may cancel out the savings achieved with the cheaper drug. Ultimately, it is the responsibility of the physician-patient-pharmacist triad to arrive at the best choice for the best patient outcome.

## Abbreviations

AADC: L-amino acid decarboxylase; API: Active pharmaceutical ingredient; DA: Dopamine; DDC: L-Dopa decarboxylase; HCl: Hydrochloride; L-dopa: Levodopa; PD: Parkinson’s Disease.

## Competing interests

FA and JPT are employees at Roche. UA and IA received consultant fees.

## Authors’ contributions

FA and JPT conceived and designed the study, and collected the data. UA and IA analysed and interpretated the data. All authors were involved in drafting and revising the manuscript critically for intellectual content and gave final approval of the version to be published.

## Author’s information

UA is an independent senior scientist working for different companies as a consultant. IA is a researcher at the University of Basel specialised in adherence to medication and in guidelines developement for community pharmacies. She is a lecturer and member of several pharmacy and pharmacology societies.

## Pre-publication history

The pre-publication history for this paper can be accessed here:

http://www.biomedcentral.com/2050-6511/14/24/prepub
